# Supplementary material for: Targeting the latent human cytomegalovirus reservoir for T-cell-mediated killing with virus-specific nanobodies
Source: Nat Commun. 2021 Jul 21;12:4436. doi: 10.1038/s41467-021-24608-5 (PMC8295288; doi:10.1038/s41467-021-24608-5)
Supplement: Supplementary file 2 — Supplementary Information [file 41467_2021_24608_MOESM2_ESM.pdf]

Supplementary Information

Targeting of the latent human cytomegalovirus reservoir for T-cell mediated killing with virus specific nanobodies

Authors:

Timo W.M. De Groof, Elizabeth G. Elder, Eleanor Y. Lim, Raimond Heukers, Nick D. Bergkamp, Ian J. Groves, Mark Wills, John H. Sinclair and Martine J. Smit

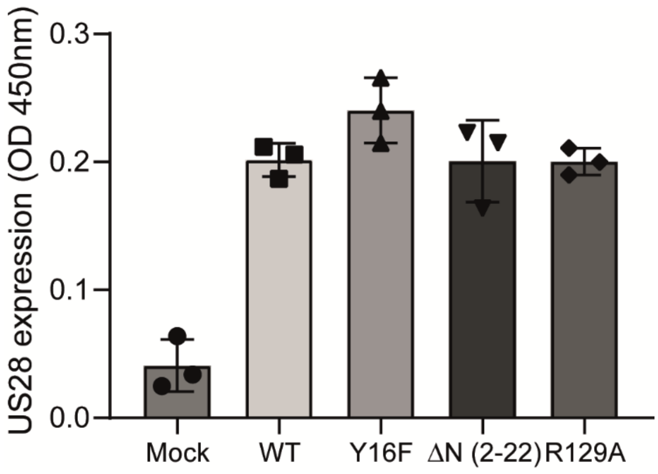

Supplementary Figure 1. Expression of US28 wildtype and mutants in transfected HEK293T cells. ELISA on HEK293T cells transfected with empty vector (Mock), vector encoding HA-US28 wildtype receptor (US28 WT), HA-US28 Y16F mutant (Y16F), HA-US28 ΔN mutant (ΔN (2-22)) or US28 R129A mutant (R129A). Receptor expression was determined by the N-terminal HA-tag and an anti-HA antibody. Representative figures from three biological replicates is shown. Data is plotted as mean ± S.D.. Source data are provided as a Source Data file.

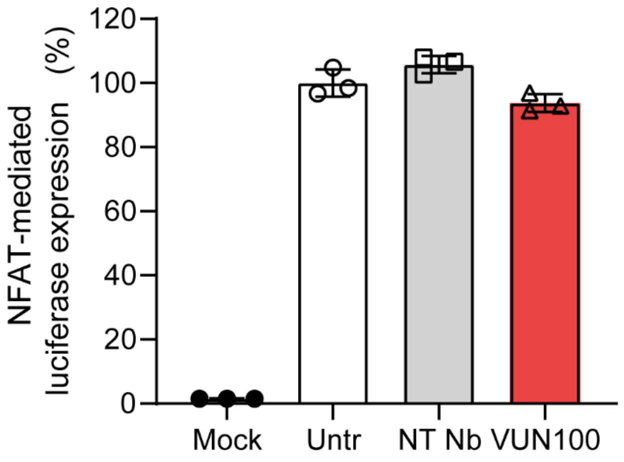

Supplementary Figure 2. Effect of high concentration VUN100 on US28-mediated NFAT activation. HEK293T cells were transfected with NFAT-luciferase reporter only (Mock) or NFAT-luciferase reporter with US28 wildtype receptor. Cells expressing the NFAT-luciferase reporter and US28 wildtype receptor were left untreated (Untr) or treated with a concentration of 1 µM of non-targeting nanobody (NT Nb) or VUN100 for 24 h prior to luminescence measurement. Data was normalized to the untreated samples. Representative figures from three biological replicates is shown. Data is plotted as mean ± S.D.. Source data are provided as a Source Data file.

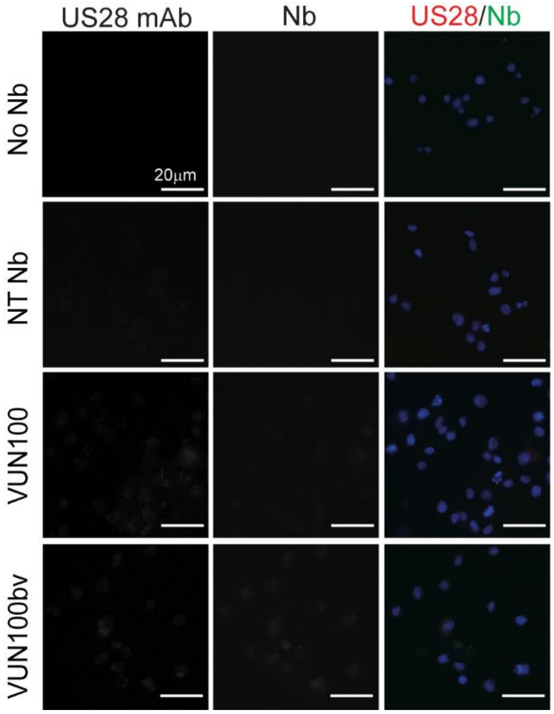

Supplementary Figure 3. Binding of nanobodies to Mock transduced THP-1 cells. Immunofluorescence microscopy of nanobody binding to mock transduced THP-1 cells. US28 staining was performed using an anti-US28 antibody (US28 mAb). Cells were incubated without nanobody (No Nb), a non-targeting nanobody (NT Nb), VUN100 or VUN100bv. Nanobody binding was detected using the Myc-tag and an anti-Myc antibody (Nb). Representative figure of 3 independent experiments is shown.

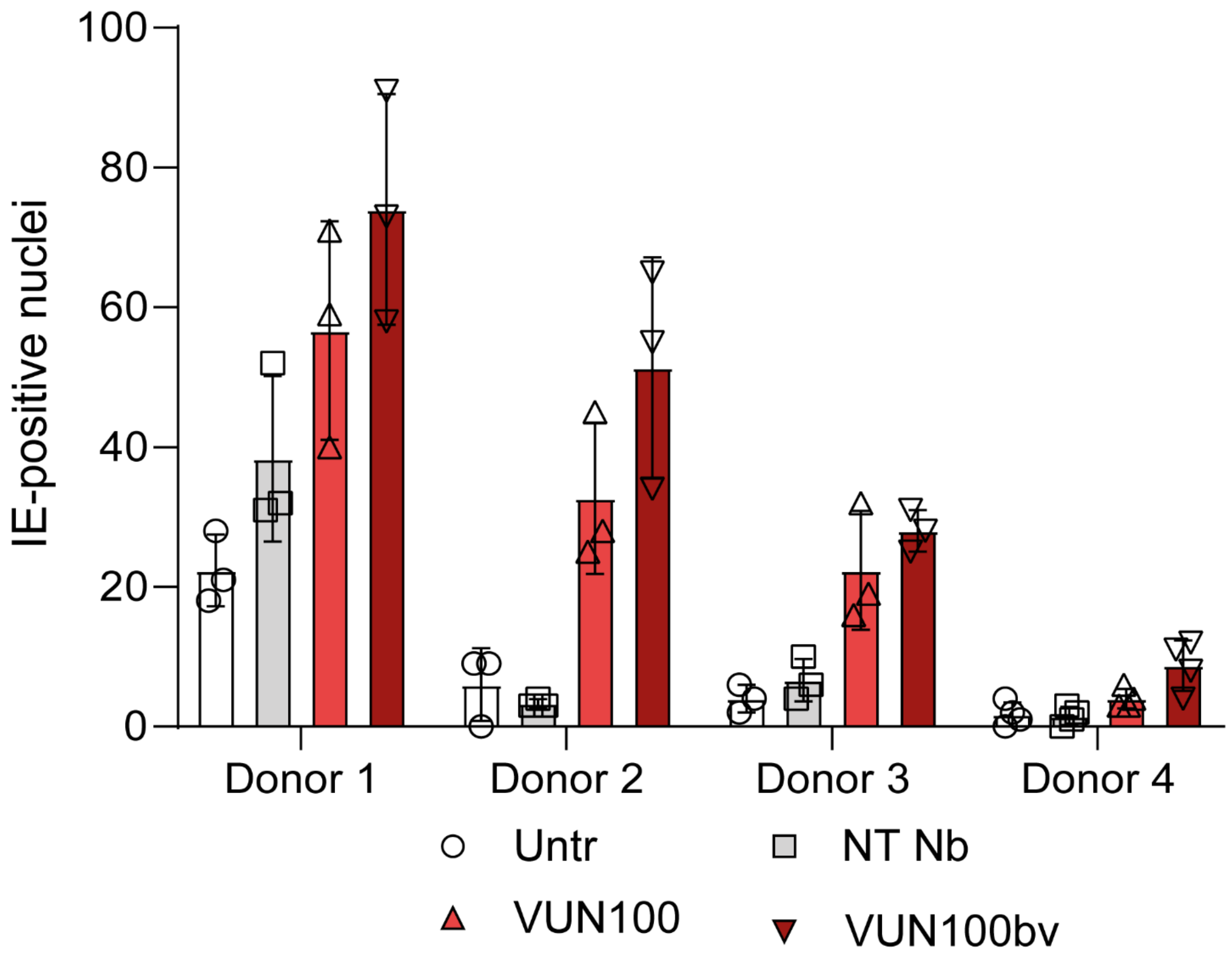

Supplementary Figure 4. Overview of immediate-early positive cells after nanobody treatment of latently infected CD14$^+$ monocytes of 4 different donors. CD14$^+$ monocytes were isolated, infected with HCMV IE2-eYFP and left untreated (Untr) or treated with a non-targeting nanobody (NT Nb), VUN100 or VUN100bv. IE-positive nuclei were counted 6 days post infection. Data, showing technical replicates, of four different donors is plotted as mean ± S.D.. Source data are provided as a Source Data file.

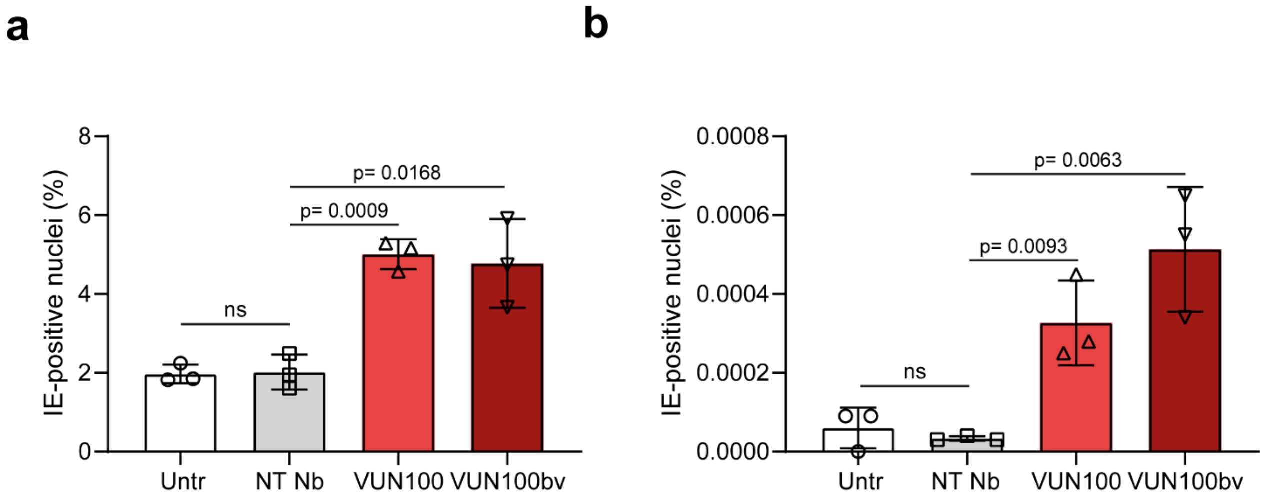

Supplementary Figure 5. Immediate-early positive cells after nanobody treatment of latently infected CD14^{+} monocytes after 2 and 6 days post infection. CD14+ monocytes were isolated, infected with HCMV IE2-eYFP and left untreated (Untr) or treated with a non-targeting nanobody (NT Nb), VUN100 or VUN100bv for 2 (**a**) and 6 (**b**) days. Six days post infection, cells were fixed and analysed for immediate early (IE)- expression using the Thermo Fisher ArrayScan system. Total numbers of cells were visualized by Hoechst staining. Representative figure, showing technical replicates, from two biological replicates is shown. All data is plotted as mean ± S.D.. For all figures, statistical analyses were performed using unpaired two-tailed t-test. ns, $p > 0.05$. Source data are provided as a Source Data file.

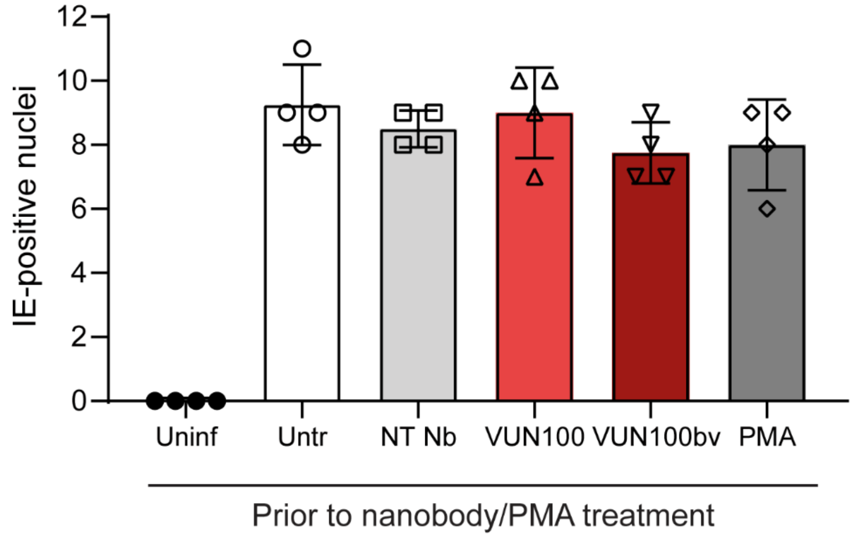

Supplementary Figure 6. Immediate-early positive CD14$^+$ monocytes 6 days post infection. CD14$^+$ monocytes were isolated and seeded. The next day, cells were uninfected (Uninf) or infected with HCMV IE2-YFP for 2 hours. IE2-positive nuclei were counted 6 days post infection prior to no treatment (Untr) or treatment with a non-targeting nanobody (NT Nb), VUN100 or VUN100bv or 20 ng/ml PMA (PMA). Representative figure, showing technical replicates, from two biological replicates is shown. Data is plotted as mean ± S.D.. Source data are provided as a Source Data file.

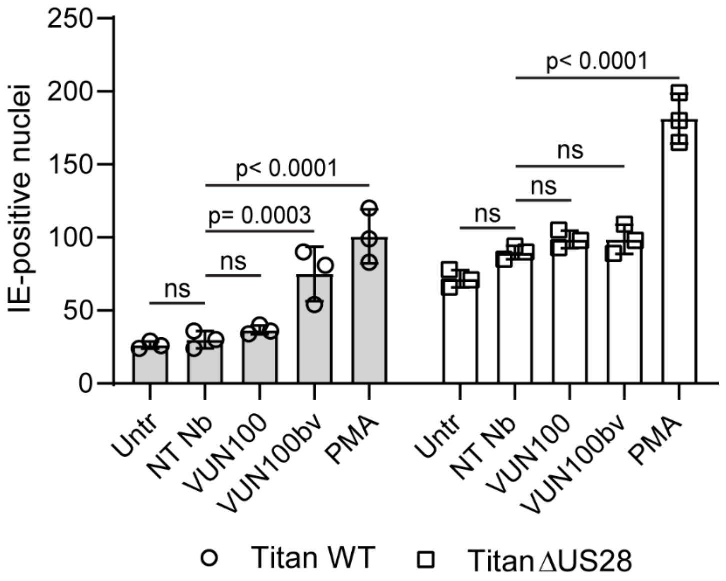

Supplementary Figure 7. Immediate-early positive CD14$^+$ monocytes 6 days post infection infected with HCMV titan WT or Titan ΔUS28 virus. CD14$^+$ monocytes were isolated and infected with HCMV Titan wildtype (Titan WT) or Titan ΔUS28 virus (white squares) for 2 hours the next day. Six days post infection, cells were untreated (Untr), treated with a non-targeting nanobody (NT Nb), VUN100, VUN100bv or 20ng/ml PMA (PMA). IE-positive nuclei were counted 3 days post infection. The results, showing technical replicates, of two biological replicates are shown. Data is plotted as mean ± S.D.. Statistical analyses were performed using unpaired two-way ANOVA with Tukey's multiple comparison test. ns, $p > 0.05$. Source data are provided as a Source Data file.

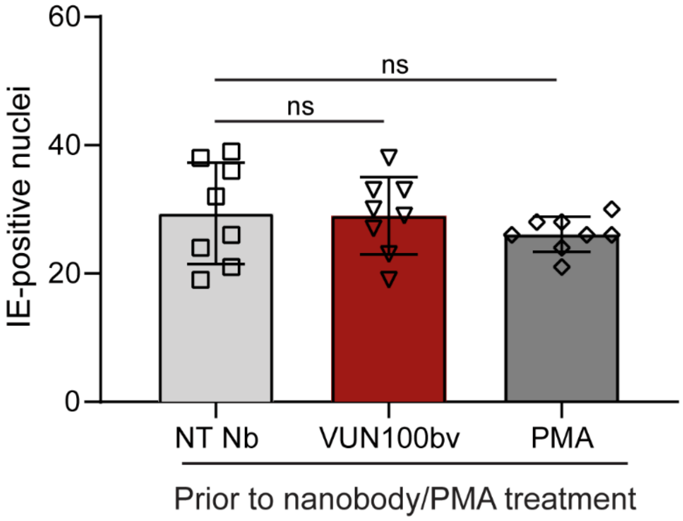

Supplementary Figure 8. Immediate-early positive CD14^{+} monocytes 6 days post infection before treatment and T-cell co-culturing. CD14^{+} monocytes were isolated and seeded. The next day, cells were infected with HCMV IE2-YFP for 2 hours. IE2-positive nuclei were counted 6 days post infection prior to treatment with a non-targeting nanobody (NT Nb), VUN100bv or 20 ng/ml PMA (PMA) and subsequent co-culturing with T-cells. Representative figure, showing technical replicates, from two biological replicates is shown. Data is plotted as mean ± S.D.. Statistical analyses were performed using unpaired two-tailed t-test. ns, $p > 0.05$. Source data are provided as a Source Data file.

Supplementary Table 1. Overview of immediate-early positive cells after nanobody treatment of latently infected CD14^{+} monocytes.

Donor	Untreated (mean + S.D.)	NT Nb (mean + S.D.)	VUN100 (mean + S.D.)	VUN100bv (mean + S.D.)
1	22 ± 4	38 ± 10	57 ± 13	74 ± 13
2	6 ± 4	3 ± 0	33 ± 9	51 ± 13
3	4 ± 2	7 ± 2	22 ± 7	28 ± 2
4	2 ± 2	2 ± 1	4 ± 1	10 ± 2

Supplementary Table 2. Overview of primers used for qPCR.

Oligo name	Oligo sequence (5' to 3')
IE72 Fwd	GTCCTGACAGAACTCGTCAAA
IE72 Rev	TAAAGGCGCCAGTGAATTTTTCTTC
UL44 Fwd	TACAACAGCGTGTCGTGCTCCG
UL44 Rev	GGCGTAAAAAACATGCGTATCAAC
GAPDH Fwd	TGCACCACCAACTGCTTAGC
GAPDH Rev	GGCATGGACTGTGGTCATGAG
pp150/UL32 Fwd	GGTTTCTGGCTCGTGGATGTCG
pp150/UL32 Rev	CACACAACACCGTCGTCCGATTAC
US11 Fwd	TACTCCGAAACATCGGGCAG
US11 Rev	CGCGGGTAGTATGCCTGAAT
GAPDH promoter Fwd	CGGCTACTAGCGGTTTTACG
GAPDH promoter Rev	AAGAAGATGCGGCTGACTGT
UL44 promoter Fwd	AACCTGAGCGTGTTTGTG
UL44 promoter Rev	CGTGCAAGTCTCGACTAAG
