# Supplementary material for: Targeting the latent human cytomegalovirus reservoir for T-cell-mediated killing with virus-specific nanobodies
Source: Nat Commun. 2021 Jul 21;12:4436. doi: 10.1038/s41467-021-24608-5 (PMC8295288; doi:10.1038/s41467-021-24608-5)
Supplement: Supplementary file 3 — Reporting Summary [file 41467_2021_24608_MOESM3_ESM.pdf]

nature research

Corresponding author(s): Martine J. Smit

Last updated by author(s): May 24, 2021

Reporting Summary

Nature Research wishes to improve the reproducibility of the work that we publish. This form provides structure for consistency and transparency in reporting. For further information on Nature Research policies, see Authors & Referees and the Editorial Policy Checklist .

Statistics

For all statistical analyses, confirm that the following items are present in the figure legend, table legend, main text, or Methods section.

n/a	Confirmed	
☐	☒	The exact sample size (*n*) for each experimental group/condition, given as a discrete number and unit of measurement
☐	☒	A statement on whether measurements were taken from distinct samples or whether the same sample was measured repeatedly
☐	☒	The statistical test(s) used AND whether they are one- or two-sided<br>*Only common tests should be described solely by name; describe more complex techniques in the Methods section.*
☒	☐	A description of all covariates tested
☒	☐	A description of any assumptions or corrections, such as tests of normality and adjustment for multiple comparisons
☐	☒	A full description of the statistical parameters including central tendency (e.g. means) or other basic estimates (e.g. regression coefficient) AND variation (e.g. standard deviation) or associated estimates of uncertainty (e.g. confidence intervals)
☐	☒	For null hypothesis testing, the test statistic (e.g. *F*, *t*, *r*) with confidence intervals, effect sizes, degrees of freedom and *P* value noted<br>*Give P values as exact values whenever suitable.*
☒	☐	For Bayesian analysis, information on the choice of priors and Markov chain Monte Carlo settings
☒	☐	For hierarchical and complex designs, identification of the appropriate level for tests and full reporting of outcomes
☒	☐	Estimates of effect sizes (e.g. Cohen's *d*, Pearson's *r*), indicating how they were calculated

Our web collection on statistics for biologists contains articles on many of the points above.

Software and code

Policy information about availability of computer code

Data collection	No software was used for data collection
Data analysis	• Data analysis was performed and graphs were plotted with GraphPad Prism version 8.0 for Windows, GraphPad Software, la Jolla California USA, www.graphpad.com<br><br>• Automated analysis of IE positive cells was carried out using ArrayScan XTI instrument using the Target Activation experimental tool in Thermo Scientific™ Store™ Express Image and Database Management Software

For manuscripts utilizing custom algorithms or software that are central to the research but not yet described in published literature, software must be made available to editors/reviewers. We strongly encourage code deposition in a community repository (e.g. GitHub). See the Nature Research guidelines for submitting code & software for further information.

Data

Policy information about availability of data

All manuscripts must include a data availability statement. This statement should provide the following information, where applicable:

- Accession codes, unique identifiers, or web links for publicly available datasets
- A list of figures that have associated raw data
- A description of any restrictions on data availability

All data associated with this study are present in the paper or in the Supplementary Materials. The source data are provided as a Source Data file.

Field-specific reporting

Please select the one below that is the best fit for your research. If you are not sure, read the appropriate sections before making your selection.

- [x] Life sciences
- [ ] Behavioural & social sciences
- [ ] Ecological, evolutionary & environmental sciences

For a reference copy of the document with all sections, see nature.com/documents/nr-reporting-summary-flat.pdf

Life sciences study design

All studies must disclose on these points even when the disclosure is negative.

Sample size	no samples size was calculated beforehand. For the validation of the nanobody, each experiment is consisting of two or four technical replicates of three to four biological replicates. For the virus experiments, three to six technical replicates of two to four different donors has been used. No outliers were removed from the experiments. Sample sizes were chosen based on previous published research.
Data exclusions	No data was excluded.
Replication	For all experiments, replication of the phenotype or observation was successful. For the validation of the nanobody, each experiment is has been performed three times independently. For the virus experiments, two to four independent experiments were performed. Experimental replicates have been stated in the manuscript.
Randomization	During all experiments, samples were randomized.
Blinding	Acquisition and analysis of data was performed blind.

Reporting for specific materials, systems and methods

We require information from authors about some types of materials, experimental systems and methods used in many studies. Here, indicate whether each material, system or method listed is relevant to your study. If you are not sure if a list item applies to your research, read the appropriate section before selecting a response.

Materials & experimental systems

n/a	Involved in the study	
	x	Antibodies
	x	Eukaryotic cell lines
x		Palaeontology
x		Animals and other organisms
	x	Human research participants
x		Clinical data

Methods

n/a	Involved in the study	
x		ChIP-seq
x		Flow cytometry
x		MRI-based neuroimaging

Antibodies

Antibodies used

- Mouse anti-Myc-tag antibody. Supplier name: Cell Signaling Technology. Catalog number: 2276S. Clone name: 9B11. Lot number: 24. Dilution: 1:1000
- Polyclonal rabbit anti-US28 antibody. Supplier name: Custom generated by Covance. Dilution: 1:1000
- Anti-mouse IgG (H+L)-HRP conjugate antibody. Supplier name: Bio-Rad. Catalog number: 170-6516. Lot number: L005680A Dilution: 1:10000
- Anti-rabbit IgG (H+L)-HRP conjugate antibody. Supplier name: Bio-Rad. Catalog number: 170-6515. Lot number: L005679A. Dilution: 1:10000
- Alexa Fluor® 488- and Alexa Fluor® 546-conjugated anti-mouse and anti-rabbit antibodies. Supplier name: Thermo Fisher Scientific. Catalog numbers: A11001 and A11010 respectively. Lot numbers: 2090562 and 1813035. Dilution: 1:1000
- Mouse-anti-actin antibody. Provider: Sigma-Aldrich. Catalog number: A5316. Clone AC-74. Lot number: 059M4770V. Dilution: 1:2000
- Mouse-anti-IE antibody. Provider: Argene. Catalog number: 11-003. Lot number: 1099280. Dilution: 1:1000
- Rat-anti-HA antibody. Provider: Roche. Catalog number: 11867423001. Clone 3F10. Lot number: 42155800. Dilution: 1:1000
- Anti-Rat-IgG-HRP conjugate antibody. Provider: Thermo Scientific, Pierce. Catalog number: 31470 Lot number: RF236103. Dilution: 1:1000
- Mouse-anti-IFI16 antibody. Provider: Santa Cruz. Catalog number: SC-8023 Lot number: G2915. Dilution: 1:500

Validation

- Mouse anti-Myc-tag antibody. Supplier name: Cell Signaling Technology. Cata log number: 2276S. Clone name: 9B11. Lot number: 24

The antibodies were validated for immunoprecipitation and immunofluorescence microscopy by Cell Signaling Technology. We

have validated them by immunofluorescence microscopy on cells transfected with the wild type US28 receptor upon incubation with Myc-tagged nanobodies (Heukers et al, Oncogene, 2018). Cells incubated without Myc-tagged nanobodies served as negative control for specific binding of the anti-Myc-tag antibody (data not shown in manuscript).
• Polyclonal rabbit anti-US28 antibody. Supplier name: Custom generated by Covance.
Method of validation:
The antibodies were previously validated by Bongers et al., J Clin Invest, 2010 and Heukers et al, Oncogene, 2018.
• Anti-mouse IgG (H+L)-HRP conjugate antibody. Supplier name: Bio-Rad.
Catalog number: 170-6516. Lot number: L005680A
Method of validation:
Described by: Knapp et al., Elsevier/North Holland Biomedical Press, 1978
• Anti-rabbit IgG (H+L)-HRP conjugate antibody. Supplier name: Bio-Rad.
Catalog number: 170-6515. Lot number: L005679A
Method of validation:
Described by: Knapp et al., Elsevier/North Holland Biomedical Press, 1978
• Alexa Fluor® 488- and Alexa Fluor® 546-conjugated anti-mouse and anti-rabbit antibodies. Supplier name: Thermo Fisher Scientific. Catalog numbers: A11001and A11010 respectively. Lot numbers: 2090562 and 1813035
Method of validation:
We have validated them on immunofluorescence microscopy cells US28 overexpressing cells. Non-expressing served as control.
• Mouse-anti-actin antibody. Provider: Sigma-Aldrich. Catalog number: A5316. Clone AC-74. Lot number: 059M4770V
Method of validation:
The antibody was previously validated by others including Kornblum et al, Nature Genetics, 2013.
• Mouse-anti-IE antibody. Provider: Argene. Catalog number: 11-003. Lot number: 1099280
Method of validation:
The antibodies were validated by others including Businger et al, Nature Microbiology, 2019.
• Rat-anti-HA antibody. Provider: Roche. Catalog number: 11867423001. Clone 3F10. Lot number: 42155800
Method of validation:
The antibodies were validated by Roche via western blot and we have validated specificity previously (De Groof et al, Molecular Pharmaceutics, 2019).
• Anti-Rat-IgG-HRP conjugate antibody. Provider: Thermo Scientific, Pierce. Catalog number: 31470 Lot number: RF236103
Method of validation:
The antibodies were validated by Thermo Fischer Scientific via western blot and immunohistochemistry.
• Mouse-anti-IFI16 antibody. Provider: Santa Cruz. Catalog number: SC-8023 Lot number: G2915
The antibodies were validated previously by us in a previous paper Elder et al, mBio, 2019.

Eukaryotic cell lines

Policy information about cell lines

Cell line source(s)	HEK293T: derived from fetal human kidney. Obtained from ATCC.<br>THP-1: derived from human peripheral blood. Obtained from European Collection of Authenticated Cell (ECAC)<br>hfff1: derived from human foreskin. Obtained from European Collection of Authenticated Cell (ECAC)
Authentication	none of the cell lines were authenticated
Mycoplasma contamination	All cell lines were mycoplasm negative, as determined via PCR by Microbiome (Amsterdam, the Netherlands), using thw R&D Mycoprobe Mycoplasm detection kit and DAPI staining.
Commonly misidentified lines<br>(See ICLAC register)	No commonly misidentified cell lines were used.

Human research participants

Policy information about studies involving human research participants

Population characteristics	Monocytes isolated from leukapherisis cones from UK NHS blood and transplant are anonymised so population characteristics were unknown. For PBMC isolation from arm bleeds, this was carried out by venous puncture of healthy donors who were chosen solely on the basis of HCMV seropositive status with no other Tier 1 reportable items according to the Biospecimen Reporting for Improved Study Quality guidelines.
Recruitment	Leukapherisis cones were obtained from the UK NHS Blood and Transplant from consenting donors. These were anonymised so population characteristics were unknown. We know of no bias of selection. PBMC from arm bleeds were performed on healthy donors chosen solely on the basis of HCMV seropositive status.
Ethics oversight	Human samples were obtained under ethical approval from Cambridgeshire 2 Research Ethics Committee (REC reference 97/092) conducted in accordance with the Declaration of Helsinki.

Note that full information on the approval of the study protocol must also be provided in the manuscript.
